# Impact of flow cytometry-based sorting on microRNA signature of extracellular vesicles derived from mesenchymal stromal cells: a proof-of-concept study

**DOI:** 10.20517/evcna.2025.130

**Published:** 2026-01-21

**Authors:** Enrico Ragni, Monica Romanò, Michela Maria Taiana, Daniela Boselli, Raffaella Pini, Donatella Biancolini, Caterina Visconte, Dejan Lazarevic, Chiara Villa, Laura de Girolamo

**Affiliations:** ^1^Laboratorio di Biotecnologie Applicate all’Ortopedia, IRCCS Ospedale Galeazzi - Sant’Ambrogio, Milano I-20157, Italy.; ^2^Experimental Imaging Center, FRACTAL, Flow cytometry Resource, Advanced Cytometry Technical Applications Laboratory, IRCCS Ospedale San Raffaele, Milano I-20132, Italy.; ^3^Center for Omics Sciences, IRCCS Ospedale San Raffaele, Milano I-20132, Italy.

**Keywords:** Extracellular vesicles, mesenchymal stromal cells, fluorescence-based sorting, microRNA profiling, osteoarthritis, regenerative medicine

## Abstract

**Aim:** This proof-of-concept study aimed to evaluate the impact of fluorescence-based sorting on the microRNA (miRNA) molecular profile of extracellular vesicles (EVs) derived from adipose-derived mesenchymal stromal cells (ASCs) (ASC-EVs), with a focus on osteoarthritis (OA) as a model disease.

**Methods:** ASCs from five human donors were characterized by flow cytometry and cultured to collect conditioned media. EV isolation was performed by fluorescence-based sorting using a high-sensitivity cell sorter calibrated for particles ≥ 100 nm. In both ASC-EVs and sorted ASC-EVs (sASC-EVs), EV and MSC markers were analysed by flow cytometry, while size and morphology were assessed via nanoparticle tracking analysis and electron microscopy. Small RNA sequencing was used to profile miRNAs, followed by differential expression and functional enrichment analyses.

**Results:** EVs displayed comparable size distributions and surface marker profiles before and after sorting. In contrast, small RNA sequencing revealed a marked reduction in the number of detectable miRNAs in sASC-EVs relative to EVs isolated by standard ultracentrifugation (285 *vs.* 749). Among these, 271 miRNAs were shared between groups, exhibiting a strong correlation in relative abundance and functional enrichment in angiogenesis, inflammation modulation and gene silencing pathways. Nevertheless, differential expression analysis identified 32 upregulated and 6 downregulated miRNAs in sASC-EVs, with 14 miRNAs detected exclusively after sorting. Despite these transcriptional differences, the overall balance between protective and detrimental OA-related miRNAs remained positive and was preserved across both ASC-EVs and sASC-EVs.

**Conclusion:** While sorting reduces EV-associated miRNA diversity, it retains core functional signals. The difference in recovered miRNAs may be due to lower EV recovery, exclusion of miRNA-bound non-vesicular particles or loss of small EVs undetectable by fluorescence-based sorting techniques. Overall, these preliminary findings highlight a trade-off between purity and complexity, underscoring the importance of optimizing EV isolation protocols for clinical applications.

## INTRODUCTION

In recent years, biological activity of mesenchymal stromal cells (MSCs) has been ascribed to their anti-inflammatory and immunodulatory potential, strictly linked to the release of soluble factors and extracellular vesicles (EVs)^[1]^. MSC-derived EVs (MSC-EVs), carrying functional molecules such as growth factors, cytokines and microRNAs (miRNAs), play beneficial roles in tissue repair by promoting angiogenesis, cell migration, proliferation, extracellular matrix remodeling and reducing inflammation^[2]^. Looking ahead, MSC-EVs may thus offer a cell-free therapeutic alternative, capturing the beneficial effects of MSCs while potentially overcoming challenges related to cell viability, delivery and immune compatibility^[3]^. In this perspective, several clinical trials are registered for the use of MSC-EVs^[4]^.

For further development of this clinical option, a reliable and reproducible EV production pipeline, along with thorough characterization, is critical for understanding and harnessing their therapeutic potential. In the vast majority of published clinical studies, ultracentrifugation - considered for several years the “gold standard” for EV isolation - is still used most often^[4]^. Despite its ease, clinical translation of this technique to large‐scale manufacturing is not optimal regarding batch‐to‐batch consistency due to intermediate recovery and specificity related to the possible co‐isolation of aggregates and/or unwanted non‐EV nanosized materials with sedimentation coefficient as EVs^[5]^. For these reasons, several alternative techniques have been developed over the years, with both strengths and pitfalls^[6]^. Among them, most recently, flow cytometry-based EV sorting emerged as a potential innovative approach^[7]^. Despite the widespread use of cell sorting, EV sorting remains technically challenging, as most EVs fall below the detection threshold of conventional flow cytometers. Reliable EV analysis and sorting by flow cytometry require high-resolution instrumentation and careful optimization of parameters - such as threshold, nozzle size, and sheath/sample pressures - to minimize background noise, swarm detection, and false positives^[8]^. Fluorescent labeling with dyes or antibodies (Abs) improves EV identification based on size, light scattering and fluorescence. However, the small size and weak signals of EVs, together with artifacts from unbound dyes or protein aggregates, continue to limit sensitivity and reproducibility^[9]^. In this frame, carboxyfluorescein succinimidyl ester (CFSE) represents a promising fluorescence-based sorting strategy^[10]^. CFSE selectively stains structures with preserved membrane integrity^[11]^. In its non-fluorescent, esterified form, CFSE diffuses across intact membranes and is subsequently hydrolyzed by esterases, generating a fluorescent derivative that retains an active succinimidyl ester moiety^[12]^. This reactive group covalently binds to primary amines on luminal and membrane-associated proteins, producing stable and long-lasting fluorescence. As a result, only structurally intact EVs are efficiently labeled, whereas disrupted or fragmented vesicles remain largely unstained. This property allows reliable discrimination of intact EV populations in functional and analytical studies and provides a robust strategy for isolating particles with high purity through fluorescence-based sorting.

Under these premises, our group developed a CFSE-based sorting protocol for adipose MSC (ASC)-EVs^[13]^. This was achieved by optimizing sorting conditions to minimize stress on the EVs, including adjusting the instrument settings to reduce sorting pressure through the selection of an appropriate nozzle size. In this way, sorting can be envisioned either as a standalone purification procedure or as an initial step combined with concentration methods, minimizing the co-retrieval of contaminants. In the present report, we characterized the EVs before and after sorting and compared their miRNA cargo through small RNA sequencing, focusing on osteoarthritis (OA) as a model disease due to recent MSC-EV applications in this field (NCT06466850). This proof-of-concept study provides a basis for advancing EV sorting as a reliable and scalable method for clinical-grade MSC-EV isolation, supporting future therapeutic applications for regenerative medicine.

## METHODS

### Ethics statement

All human-derived materials used in this study were purchased from a commercial vendor (Biopredic International, Saint-Grégoire, France) and were fully anonymized. As no patient-identifiable information was involved and the materials were not collected specifically for this study, ethical approval and informed consent were not required in accordance with institutional and national guidelines.

### ASC isolation and culture

Adipose material from five healthy female donors (36 ± 3 years old) was processed as previously reported^[14]^. Briefly, after 30 min of digestion at 37 °C with type I collagenase (Worthington Biochemical Co., Lakewood, NJ, USA), tissues were filtered through a 100 μm cell strainer, centrifuged at 1,000 × *g* for 5 min at room temperature (RT), and the pellets were suspended in alpha minimum essential medium (α-MEM) supplemented with 10% fetal bovine serum (FBS) before seeding at 5 × 10^3^ cells/cm^2^ (37 °C, 5% CO_2_, 95% humidity). ASCs were selected by plastic adherence.

### ASC characterization by flow cytometry

ASCs at passage 3 were analyzed. Abs used included anti-CD73-PE (REA804, Miltenyi Biotec, Bergisch Gladbach, Germany), CD90-FITC (fluorescein isothiocyanate; REA897, Miltenyi), CD31-PerCP-Vio700 (REA730, Miltenyi), CD44-PE (REA690, Miltenyi), and CD45-FITC (REA747, Miltenyi). Cells were stained for 30 min at 4 °C in the dark, washed with fluorescence-activated cell sorting (FACS) buffer, and analyzed by flow cytometry using a CytoFLEX flow cytometer (Beckman Coulter, Fullerton, CA, USA), with at least 30,000 events collected.

### Secretome collection

At passage 3 and 90% cell confluence, ASCs were washed, detached and suspended (37 °C, 5% CO_2_, 95% humidity) in α-MEM without FBS, ideally 1 mL per 1 × 10^6^ ASCs. After 96 h, the conditioned medium was collected. Floating cells and debris were removed by serial centrifugation at 4 °C: 400 × *g* for 10 min, 1,000 × *g* for 10 min, 2,000 × *g* for 10 min and twice 4,000 × *g* for 10 min. The supernatant was filtered through a 0.22 μm filter to remove the majority of remaining particles larger than 220 nm. The conditioned medium was used immediately or stored for a maximum of one night at 4 °C or frozen at -80 °C if further steps were not performed within 24 h.

### EV staining

EV-containing conditioned media were either freshly processed (within 24 h) or thawed from -80 °C storage and brought to 37 °C prior to staining. EVs were labeled with 10 µM CFSE [5(6)-Carboxyfluorescein diacetate N-succinimidyl ester; Merk Life Science S.r.l., Milano, Italy], prepared fresh as a 5 mM stock solution in dimethyl sulfoxide (DMSO) and incubated for 1 h at 37 °C in the dark. Following staining, samples were concentrated and washed using centrifugal ultrafiltration units with a 100 kDa molecular weight cutoff (Amicon® Ultra, MWCO 100 kDa; Merk Life Science S.r.l.). A maximum of 15 mL per run was centrifuged at 4,000 × *g* to reduce volume to 500 µL. Two sequential washes with 14 mL PBS were performed to remove unbound dye. Approximately 500 µL of concentrated, CFSE-labeled EVs were recovered and processed for downstream applications.

### Cell sorter calibration

Instrument (MoFlo Astrios EQ, Cell Sorter; Beckman Coulter, Brea, CA, USA), equipped with Blue 488 nm, Yellow-Green 561 nm, Violet 405 nm, and Red 640 nm, was calibrated as previously described^[13]^. The choice of the instrument is due to its jet-in-air system, which interrogates particles with lasers in air, thereby reducing sample stress. In addition, the presence of an obscuration bar minimizes background optical noise and provides a reference signal that facilitates the detection of events close to the instrument’s sensitivity limit. Finally, the dual-path Forward Scatter (FSC) configuration with two masks enables improved discrimination between small and large particles. Briefly, after cell sorter set-up, side scatter (SSC) and FSC parameters were calibrated using VER01A (189 nm) and VER01B (374 nm) Verity Shells hollow organo-silica beads (Exometry, Amsterdam, The Netherlands), which exhibit light scattering properties and refractive indices more similar to EVs than standard polystyrene beads. Background was assessed using 0.22 µm-filtered PBS, and instrument thresholds were optimized to minimize noise without compromising detection of the smallest beads. Sorting conditions were optimized to minimize mechanical stress on EVs. Instrument was optimized by reducing the sorting pressure associated with the nozzle size (70 µm nozzle with a lower pressure of 35 psi instead of the standard 60 psi typically applied for this nozzle size).

### EVs acquisition, sorting and purity check

The previously published procedure was followed^[13]^. Briefly, the instrument background was first established by acquiring 0.22 µm-filtered PBS on a SSC *vs.* FITC log plot at low flow rate (< 2,000 events/s) to minimize electronic noise. Unstained conditioned medium was used to assess background fluorescence (~5,000 events/s), and CFSE-stained PBS served as a negative control to confirm specificity. CFSE-stained EVs were acquired and gated into CFSE-positive and CFSE-negative populations using fluorescence and scatter parameters. Gating strategy was based on comparison to unstained and PBS controls. Sorting was conducted using a customized protocol with enabled parameters: 488-nm forward scatter 1 (FSC1), 488-nm forward scatter 2 (FSC2), 488-nm side scatter (SSC), and 488-nm/513/26 channel (CFSE). Three dot plots were used to define sorting gates: FSC1 *vs.* SSC, FSC2 *vs.* SSC and CFSE *vs.* SSC. Sorting was performed in Purify Mode with a 1-2 drop envelope to ensure precise collection of gated events. After verifying the test stream and stable flow rate, sorting was initiated and parameters were saved. Post-sorting, samples (5 µL diluted in 100 µL 0.22 µm-filtered PBS) were re-analyzed to confirm purity. Both CFSE-positive and CFSE-negative populations were recorded to assess gating accuracy and sorting efficiency.

### TEM analysis

Pre-sorted samples were diluted in 0.22 µm-filtered PBS to allow comparison with post-sorted samples based on the number of nanoparticles per field. Briefly, samples were deposited onto formvar-carbon-coated grids and incubated at RT for 10 min. Excess liquid was gently blotted using filter paper. Negative staining was performed with 2% aqueous uranyl acetate for 10 min, followed by blotting to remove the excess stain. Grids were air-dried at RT before imaging. Transmission electron microscopy (TEM) was conducted using a TALOS L120C instrument (Thermo Fisher Scientific, Waltham, MA, USA) operating at 120 kV.

### NTA analysis

Pre-sorted samples were diluted in 0.22 µm-filtered PBS to enable comparison with post-sorted samples based on the concentration measured by nanoparticle tracking analysis (NTA). Briefly, samples were analyzed using the NanoSight NS300 system (NanoSight Ltd., Amesbury, UK). For each sample, five 60 s videos were recorded. Data were processed with NTA software to obtain particle concentration and high-resolution size distribution profiles.

### EVs characterization by flow cytometry

Pre-sorted samples were diluted in 0.22 µm-filtered PBS to enable comparison with post-sorted samples based on the acquired events/ml. Briefly, prior to use, each Ab was centrifuged at 15,000-17,000 × *g* for 30 min at 4 °C to remove aggregates that may cause false-positive signals. Then, Abs were filtered through separate 0.22 µm centrifugal filter units at 1,000 × *g* and 4 °C until the entire volume passed through and no liquid remained on the filter surface. Samples, already CFSE-labeled, were divided into 100 μL aliquots. One aliquot was left unstained and the other aliquots were separately incubated for 30 min at 4 °C in the dark with 5 μL of allophycocyanin (APC)-conjugated Abs: anti-CD9 (312107, BioLegend Europe B.V., Amsterdam, The Netherlands), CD63 (353007, BioLegend), CD81 (349509, BioLegend), CD90 (328113, BioLegend), or CD73 (344005, BioLegend). One-hundred μL aliquots of PBS were similarly processed for Ab background control. After staining, 200 μL of 0.22 µm-filtered PBS were added to each sample. Acquisition and recording were performed with a low flow rate (10 µL per min) using a CytoFLEX flow cytometer. All sample volumes were acquired. Instrument settings were established using Megamix-Plus SSC + FSC reference beads (Biocytex, Marseille, France), composed of FITC-labeled polystyrene beads of 100, 160, 200, 240, 300, 500 and 900 nm in diameter. The FITC threshold was optimized to include 100 nm beads and smaller particles in the FITC/CFSE channel.

### EV isolation and RNA extraction

Post-sorted EVs (5-10 mL) and a volume of pre-sorted conditioned media equivalent to that used for sorting were ultracentrifuged using a protocol previously established in our laboratory, which efficiently recovers nanoparticles without selectively enriching specific subpopulations relative to the input^[15]^. Briefly, both pre-sorted and post-sorted samples were supplemented with 0.22 µm-filtered PBS up to 10 mL and centrifuged at 100,000 × *g* for 9 h at 4 °C in an Optima L-90K equipped with a Fixed-angle titanium rotor, Type 70.1 Ti (Beckman Coulter). Pellets were washed with 10 mL of 0.22 µm-filtered PBS and centrifuged under the same conditions. After carefully removing all remaining liquid, pellets were dissolved with QIAzol Lysis Reagent and total RNA extracted with miRNeasy Micro Kit (Qiagen, Hilden, Germany) following the manufacturer’s instructions. Due to the very low particle number, it was not possible to quantify RNA for sequencing, so all extracted nucleic acids were processed. RNA was stored at -80 °C until use.

### miRNAs profiling and data analysis

SMARTer smRNA (miRNA). Next-generation sequencing (NGS) libraries were prepared using the SMARTer smRNA-seq Kit (Cat N° 635031; TaKaRa, Mountain View, CA, USA) for Illumina. Briefly, isolated small RNAs were converted into complementary DNA (cDNA), followed by barcoding using the recommended number of polymerase chain reaction (PCR) cycles. The quality and quantity of the small RNA libraries were assessed using the Agilent 4200 TapeStation system. Libraries were then diluted and sequenced on the Illumina NovaSeq 6000 platform (Illumina, San Diego, CA, USA), targeting an average depth of 15-20 million reads per library with a read length of 100 nucleotides. Raw sequencing data were demultiplexed using the Bcl2Fastq software (Illumina, Inc., USA).

### Differential expression of ASC-EVs and sASC-EVs-derived miRNAs

Raw reads, consisting of 100-bp single-end reads, were trimmed to remove adapters and artefacts and keep only those with at least 15 nt, with cutadapt (https://cutadapt.readthedocs.io/en/stable/, v4.4)^[16]^. Then, they were aligned to the reference human genome GRCh38 (Genome Reference Consortium Human Build 38), using the bowtie aligner (v1.3.0)^[17]^, allowing only 1 mismatch in the first 20 bases and choosing the best alignment in terms of mismatch numbers and Phred quality. Expression counts at the miRNA level were estimated from mapped reads using featureCounts (v2.0.1)^[18]^ and annotated according to mature miRNA annotation on miRbase (https://www.mirbase.org/, gff3 v22.1)^[19]^, allowing fractional counting of multi-mapped and multi-overlapping reads.

Downstream analysis was performed in the R environment (https://www.R-project.org/, v4.3.1). Out of a total of 2,031 miRNAs detected, only those considered expressed were retained. We divided samples by condition (pre-sorting, post-sorting) and chose miRNAs that expressed more than 1 RPM (reads per million mapped reads) in at least 3 samples within each condition. Exploratory and differential expression analysis was performed on the panel of miRNAs detected by post-sorting. Normalization for exploratory analysis was applied with the “regularized log” (Rlog) transformation method of the DESeq2 R package (v 1.40.2)^[20]^. The principal components were calculated by the prcomp function of the base R environment. Differential expression analysis was also performed with DESeq2, using a paired design that included patient and condition and an estimate of shrinkage with apeglm^[21]^. DESeq2 uses a rigorous normalization approach specifically designed to handle different read depths and library compositions, through its median of ratios method.

Plots were drawn using the R ggplot2 (https://ggplot2.tidyverse.org, v3.5.1)^[22]^ and ComplexHeatmap (v2.16.0)^[23]^ packages.

### Functional enrichment analysis of EV miRNAs

For functional enrichment analysis of total and differentially expressed miRNAs, the miRNA Enrichment Analysis and Annotation Tool (miEAA) was used^[24]^. miEAA is a web-based tool that facilitates the functional analysis of sets of miRNAs. Annotation for Gene Ontology (GO) and GO Biological Process (miRPathDB) was tested using false discovery rate (FDR) adjustment (Benjamini-Hochberg) for *P*-value correction, with *P*-values adjusted independently for each category. A threshold of < 0.05 was set for significance.

### Statistical Analyses

Statistical analyses were performed using the GraphPad Prism software (version 8.0.2, GraphPad, San Diego, CA, USA). For comparison data about physical and phenotypic EV features, a Shapiro-Wilk test for normal distribution was performed with alpha set at 0.05. If normality was not met, a paired Wilcoxon test (non-parametric) was performed; if normality was met, a paired t-test (parametric) was used. A significance threshold of < 0.05 was applied. For correlation of Rlog values, a nonparametric Spearman correlation was computed on non-normally distributed data, with a 95% confidence interval and a two-tailed *P*-value.

## RESULTS

### ASC-EVs and sorted ASC-EVs (sASC-EVs) characterization

**Figure 1:**
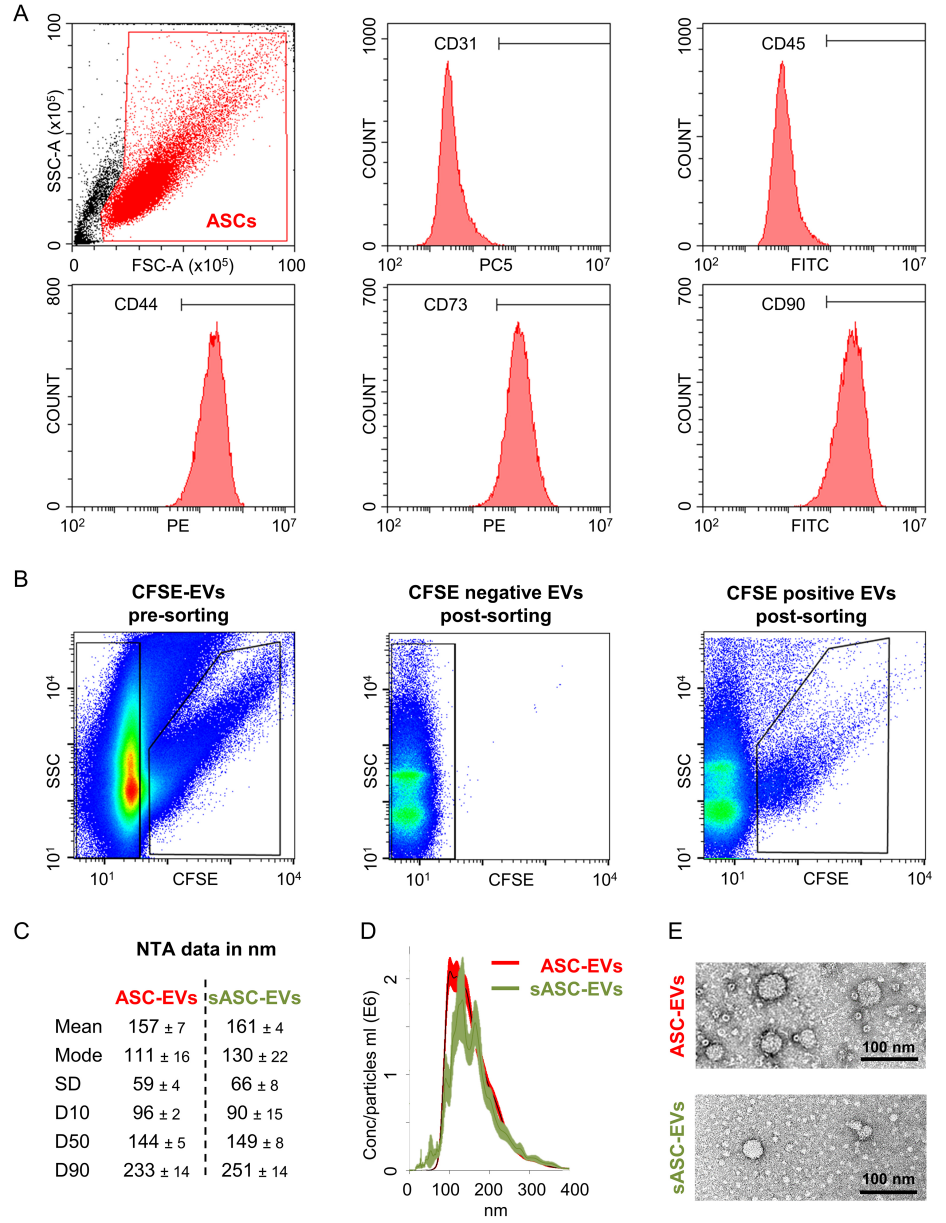
Immunophenotypic characterization of ASCs and biophysical characterization of derived EVs. (A) Flow cytometry analysis of ASCs demonstrating a typical MSC immunophenotype, with strong expression (> 95% positive cells) of canonical MSC markers CD44, CD73, and CD90, and minimal expression (< 5%) of hemato-endothelial markers CD31 and CD45. Representative cytograms are shown; (B) Representative SSC/CFSE dot plots illustrating the sorting strategy for ASC-EVs according to a previously published protocol. CFSE-positive events correspond to sorted sASC-EVs, whereas CFSE-negative events represent excluded particles; the mean recovery of sASC-EVs across five independent experiments was 10% ± 3%; (C) Size distribution of ASC-EVs and sASC-EVs determined by NTA, showing comparable mean particle sizes (ASC-EVs: 157 ± 7 nm; sASC-EVs: 161 ± 4 nm) and median diameters (D50: 144 ± 5 nm and 149 ± 8 nm, respectively); (D) NTA-derived particle concentration and size distribution profiles of ASC-EVs and sASC-EVs, demonstrating overlapping distributions and no statistically significant differences between preparations. Data are presented as mean ± SD, *n* = 5 independent experiments; (E) Representative transmission electron microscopy images from one donor confirming the presence of vesicular structures with typical EV morphology in both ASC-EVs and sASC-EVs preparations. ASCs: Adipose-derived stromal cells; MSC: mesenchymal stromal cell; EVs: extracellular vesicles; sASC: sorted adipose-derived stromal cell; CD: cluster of differentiation; SSC: side scatter; CFSE: carboxyfluorescein succinimidyl ester; NTA: nanoparticle tracking analysis; D50: median particle diameter; SD: standard deviation.

As expected, adipose-derived stromal cells (ASCs) were strongly positive (> 95%) for canonical MSC markers (CD44, CD73, CD90) and negative (< 5%) for hemato-endothelial markers (CD31, CD45) [Figure 1A].

EVs derived from ASCs (ASC-EVs) were sorted according to a previously published protocol [Figure 1B]. Across five independent repetitions, the mean recovery of sASC-EVs was 10% ± 3%. NTA revealed that ASC-EVs displayed a mean size of 157 ± 7 nm, with a median particle diameter (D50) of 144 nm ± 5 [Figure 1C]. Comparable values were obtained for sASC-EVs, with a mean size of 161 ± 4 nm and a D50 of 149 ± 8 nm [Figure 1C]. No statistically significant differences were observed between the two preparations across all measured parameters. Accordingly, both ASC-EVs and sASC-EVs displayed overlapping size distribution profiles [Figure 1D]. The presence of vesicular structures after sorting was further confirmed by TEM [Figure 1E].

**Figure 2:**
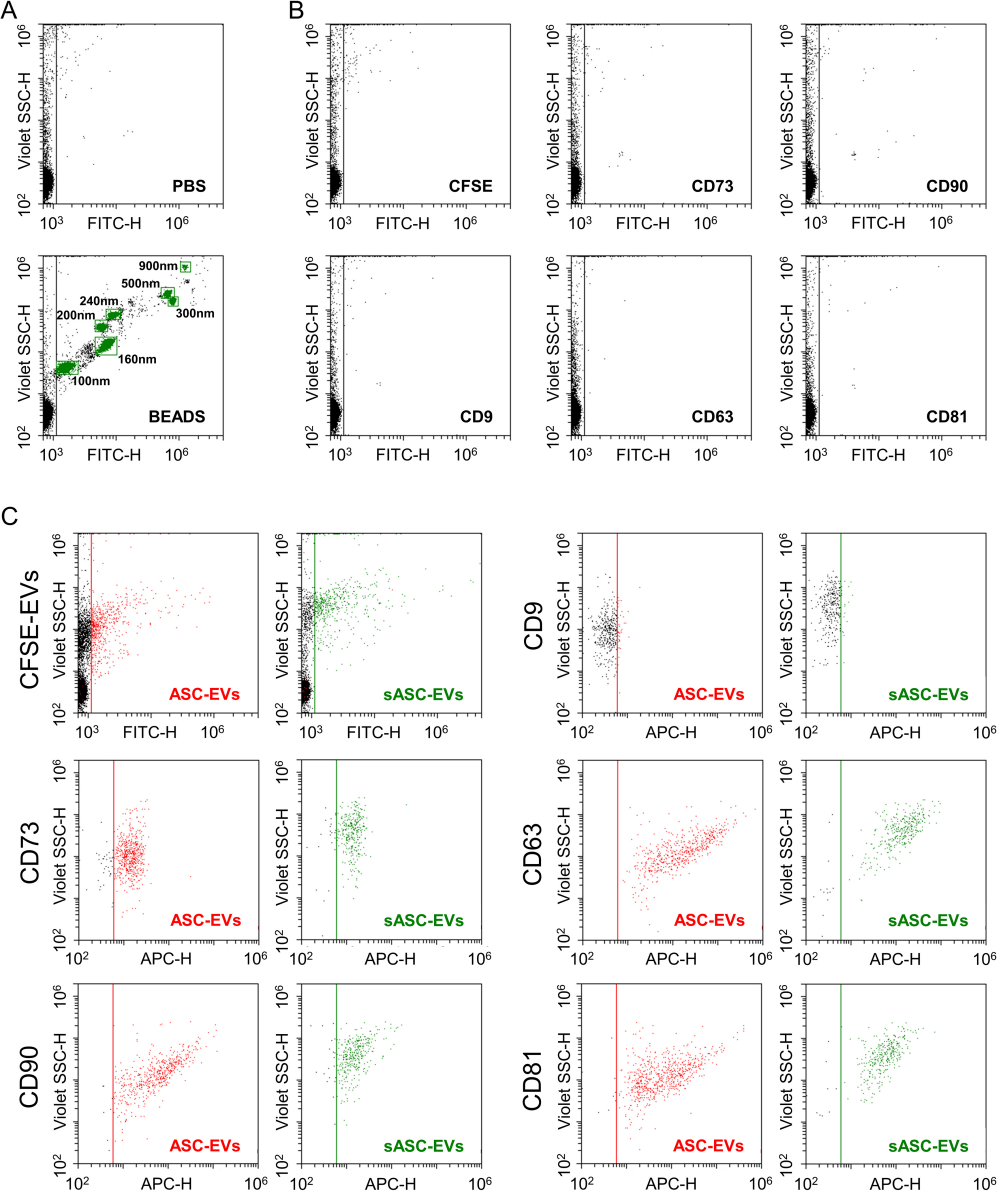
Immunophenotypic characterization of ASC-derived EVs and sASC-EVs by flow cytometry. (A) Calibration of the flow cytometer using FITC-labeled reference nanobeads with nominal diameters ranging from 100 to 900 nm, enabling reliable detection of fluorescent nanoparticles down to 100 nm and potentially below; (B) Assessment of background fluorescence in the 488 nm (FITC) channel using CFSE alone and single-antibody staining controls (anti-CD9, CD63, CD81, CD73, and CD90), demonstrating negligible background signal under the acquisition settings used for EV analysis; (C) Representative flow cytometry plots of CFSE-positive ASC-EVs and CFSE-positive sASC-EVs gated in the 488 nm (FITC) channel. Both EV preparations displayed strong expression of the canonical EV markers CD63 and CD81 and of the mesenchymal stromal cell-associated markers CD73 and CD90, whereas CD9 was only weakly detectable. Quantitatively, ASC-EVs showed low CD9 positivity (8% ± 2%) and high positivity for CD63 (98% ± 1%), CD81 (98% ± 1%), CD73 (93% ± 1%), and CD90 (100% ± 0). A highly comparable immunophenotypic profile was observed for sASC-EVs (CD9, 8% ± 1%; CD63, 93% ± 5%; CD81, 93% ± 5%; CD73, 89% ± 3%; CD90, 89% ± 8%), with no statistically significant differences between pre-sorted and sorted EVs. Representative cytograms are shown. ASC: Adipose-derived stromal cell; EVs: extracellular vesicles; sASC: sorted adipose-derived stromal cell; FITC: fluorescein isothiocyanate; CFSE: carboxyfluorescein succinimidyl ester; CD: cluster of differentiation.

Phenotypic characterization of ASC-EVs and sASC-EVs was performed by flow cytometry. The instrument was calibrated to reliably detect fluorescent particles down to 100 nm, and potentially smaller [Figure 2A]. Control experiments with the dye alone (CFSE) and with the Abs used for EV immunostaining (anti-CD9, -CD63, -CD81, -CD73, -CD90) confirmed the absence of background signal in the 488-nm channel used for nanoparticle detection [Figure 2B]. ASC-EVs exhibited minimal expression of CD9 (8% ± 2%), while showing strong positivity for EV markers CD63 (98% ± 1%) and CD81 (98% ± 1%), as well as MSC markers CD73 (93% ± 1%) and CD90 (100% ± 0) [Figure 2C]. sASC-EVs displayed a highly comparable immunophenotypic profile (CD9, 8% ± 1%; CD63, 93% ± 5%; CD81, 93% ± 5%; CD73, 89% ± 3%; CD90, 89% ± 8%), with no significant differences compared to pre-sorted preparations.

### miRNA fingerprint of ASC-EVs and sASC-EVs

**Figure 3:**
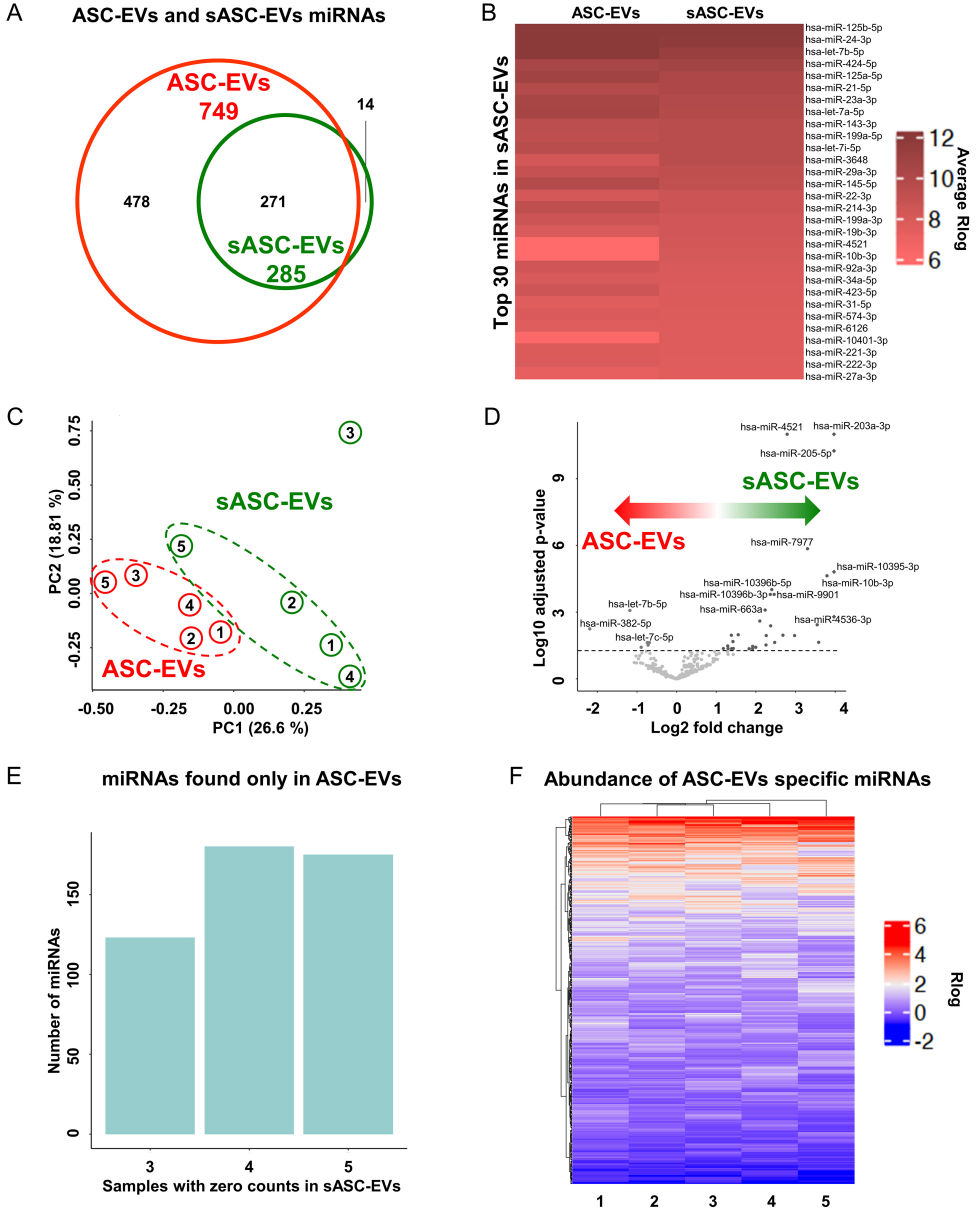
miRNA profiling of ASC-derived EVs and sASC-EVs. (A) Venn diagram summarizing miRNAs detected by NGS across five donors. A total of 749 distinct miRNAs were identified in ASC-EVs and 285 in sASC-EVs, with 271 miRNAs shared between the two groups, 478 detected exclusively in ASC-EVs, and 14 uniquely identified in sASC-EVs; (B) Heatmap of the Top-30 most abundant miRNAs ranked according to expression levels in sASC-EVs. Colors represent average miRNA expression after Rlog transformation. The majority of these highly expressed miRNAs were also among the most abundant in ASC-EVs, indicating strong concordance between pre- and post-sorted EV preparations; (C) PCA based on Rlog-transformed expression values of the 271 shared miRNAs, showing clear discrimination between ASC-EVs and sASC-EVs despite the overall high correlation in miRNA expression profiles; (D) Volcano plot depicting differential miRNA expression between sASC-EVs and ASC-EVs. The significance threshold was set at -log10 adjusted *P*-value > 1.3 (adjusted *P* < 0.05), *n* = 5 donors, identifying 38 differentially expressed miRNAs, with upregulation in sASC-EVs as the predominant effect of sorting; (E) Count distribution analysis of the 478 miRNAs detected exclusively in ASC-EVs. Bars indicate the number of miRNAs showing zero counts in 3, 4, or all 5 post-sorting sASC-EV samples, supporting their classification as absent in sASC-Evs; (F) Heatmap of ASC-EV-specific miRNAs, displaying average Rlog-transformed expression levels across donors. Color intensity reflects relative abundance, with red and blue indicating higher and lower expression, respectively, and highlighting the generally low abundance of these miRNAs (Rlog < 6). ASC: Adipose-derived stromal cell; EVs: extracellular vesicles; sASC: sorted adipose-derived stromal cell; miRNA: microRNA; NGS: next-generation sequencing; Rlog: regularized log transformation; PCA: principal component analysis.

Across the five analyzed samples, sequencing yielded an average of 101 million reads ± 63 (range: 43 M in donor 3 to 215 M in donor 5) for ASC-EVs, compared to 58 million ± 26 (range: 21 M in donor 3 to 98 M in donor 4) for sASC-EVs. The number of assigned reads was 137 ± 100 K for ASC-EVs and 18 ± 14 K for sASC-EVs. In total, 749 distinct miRNAs were identified in ASC-EVs and 285 in sASC-EVs [Supplementary Table 1]. Of these, 271 miRNAs were common to both groups, 478 were specific to ASC-EVs, and 14 were detected exclusively in sASC-EVs [Figure 3A and Supplementary Table 1].

Within the 271 shared miRNAs, expression levels showed a strong correlation between pre- and post-sorted EVs (Spearman r = 0.935). This concordance was further confirmed by analysis of the Top-30 most abundant miRNAs in sASC-EVs, which also displayed high expression in ASC-EVs [Figure 3B]. Remarkably, 80% of the Top-30 miRNAs in sASC-EVs were also represented in the Top-30 of ASC-EVs, including 19 and 18 miRNAs within the Top-20 of ASC-EVs and sASC-EVs, respectively [Table 1]. Furthermore, the 67 most abundant miRNAs, accounting for the 75th percentile of expression in both groups, exhibited highly similar expression values, with minimal Rlog values of 5.4 and 5.6 and mean Rlog values of 7.6 and 7.7 for ASC-EVs and sASC-EVs, respectively. Despite this high degree of overlap, principal component analysis (PCA) was able to discriminate between pre- and post-sorted EVs [Figure 3C]. This separation was attributable to 38 differentially expressed miRNAs, including 6 downregulated and 32 upregulated in sASC-EVs [Figure 3D and Table 2]. Overall, upregulation represented the dominant effect of sorting, with 7 miRNAs displaying > 10-fold changes, reaching up to 35.11-fold for hsa-miR-203a-3p.

**Table 1:** Top-30 miRNAs in ASC-EVs and sASC-EVs, respectively, from the shared miRNAs group

**ASC-EVs**		**sASC-EVs**	
miRNA	Rlog	miRNA	Rlog
*hsa-let-7b-5p*	*12.5*	*hsa-miR-125b-5p*	*12.1*
*hsa-miR-24-3p*	*12.2*	*hsa-miR-24-3p*	*11.8*
*hsa-miR-125b-5p*	*12.2*	*hsa-let-7b-5p*	*11.3*
*hsa-miR-125a-5p*	*10.9*	*hsa-miR-424-5p*	*10.7*
*hsa-let-7a-5p*	*10.5*	*hsa-miR-125a-5p*	*10.2*
*hsa-miR-23a-3p*	*10.5*	*hsa-miR-21-5p*	*10.1*
*hsa-miR-424-5p*	*10.5*	*hsa-miR-23a-3p*	*9.9*
*hsa-miR-21-5p*	*9.8*	*hsa-let-7a-5p*	*9.8*
*hsa-miR-145-5p*	*9.8*	*hsa-miR-143-3p*	*9.6*
*hsa-let-7i-5p*	*9.6*	*hsa-miR-199a-5p*	*9.6*
*hsa-miR-143-3p*	*9.4*	*hsa-let-7i-5p*	*9.6*
*hsa-miR-214-3p*	*9.3*	*hsa-miR-3648*	*9.6*
*hsa-miR-199a-5p*	*9.3*	*hsa-miR-29a-3p*	*9.3*
*hsa-miR-29a-3p*	*8.9*	*hsa-miR-145-5p*	*9.2*
*hsa-miR-199a-3p*	*8.6*	*hsa-miR-22-3p*	*8.6*
*hsa-miR-423-5p*	*8.5*	*hsa-miR-214-3p*	*8.6*
*hsa-miR-92a-3p*	*8.4*	*hsa-miR-199a-3p*	*8.4*
*hsa-miR-3648*	*8.3*	*hsa-miR-19b-3p*	*8.3*
*hsa-miR-22-3p*	*8.2*	hsa-miR-4521	8.3
hsa-let-7e-5p	8.1	hsa-miR-10b-3p	8.1
hsa-let-7c-5p	8.1	*hsa-miR-92a-3p*	*8.0*
*hsa-miR-19b-3p*	*8.0*	*hsa-miR-34a-5p*	*7.9*
*hsa-miR-34a-5p*	*7.9*	*hsa-miR-423-5p*	*7.8*
hsa-let-7f-5p	7.9	hsa-miR-31-5p	7.8
hsa-miR-424-3p	7.9	*hsa-miR-574-3p*	*7.7*
*hsa-miR-574-3p*	*7.9*	hsa-miR-6126	7.7
*hsa-miR-222-3p*	*7.9*	hsa-miR-10401-3p	7.7
hsa-miR-224-5p	7.8	*hsa-miR-221-3p*	*7.7*
hsa-miR-191-5p	7.8	*hsa-miR-222-3p*	*7.6*
*hsa-miR-221-3p*	*7.8*	hsa-miR-27a-3p	7.6

In italic, miRNAs present in both Top-30 lists. Rlog mean of five samples is shown. ASC-EVs: Adipose-derived stromal cell-derived extracellular vesicles; sASC-EVs: sorted adipose-derived stromal cell-derived extracellular vesicles; miRNA: microRNA; Rlog: regularized log transformation; hsa: Homo sapiens.

**Table 2:** Differentially expressed miRNAs among shared ASC-EVs and sASC-EVs

**sASC-EVs *vs.* ASC-EVs**						**sASC-EVs specific**
miRNA	FC	padj	miRNA	FC	padj	miRNA
hsa-miR-382-5p	0.22	5.63E-03	hsa-miR-663a	4.70	8.01E-04	hsa-miR-10392-5p
hsa-let-7b-5p	0.44	8.41E-04	hsa-miR-6516-5p	4.77	2.99E-02	hsa-miR-129-2-3p
hsa-let-7c-5p	0.54	3.80E-02	hsa-miR-4454	4.79	1.13E-02	hsa-miR-141-5p
hsa-let-7a-5p	0.60	2.31E-02	hsa-miR-10396b-3p	5.15	1.59E-04	hsa-miR-3130-3p
hsa-miR-424-3p	0.61	2.99E-02	hsa-miR-1843	5.18	4.13E-03	hsa-miR-3184-3p
hsa-miR-125a-5p	0.63	2.27E-02	hsa-miR-10396b-5p	5.28	9.64E-05	hsa-miR-3650
hsa-miR-138-5p	2.26	4.32E-02	hsa-miR-9901	5.50	1.59E-04	hsa-miR-376b-3p
hsa-miR-6724-5p	2.44	3.31E-02	hsa-miR-7973	5.55	2.31E-02	hsa-miR-4709-5p
hsa-miR-10401-3p	2.46	4.37E-02	hsa-miR-1248	6.42	1.09E-02	hsa-miR-4732-3p
hsa-miR-10401-5p	2.61	1.09E-02	hsa-miR-4521	6.91	4.48E-14	hsa-miR-521
hsa-miR-29b-3p	2.62	4.39E-02	hsa-miR-222-5p	7.87	1.13E-02	hsa-miR-6747-3p
hsa-miR-4449	2.68	2.09E-02	hsa-miR-7977	9.86	1.41E-06	hsa-miR-6763-3p
hsa-miR-6739-3p	2.69	4.29E-02	hsa-miR-4664-3p	11.68	3.70E-03	hsa-miR-6817-3p
hsa-miR-1246	2.94	1.04E-02	hsa-miR-4485-5p	11.99	2.27E-02	hsa-miR-9-3p
hsa-miR-10395-5p	3.55	4.40E-02	hsa-miR-10b-3p	13.87	2.32E-05	
hsa-miR-3195	3.77	4.39E-02	hsa-miR-10395-3p	20.56	1.53E-05	
hsa-miR-142-5p	3.78	3.46E-02	hsa-miR-205-5p	21.30	5.60E-11	
hsa-miR-6752-3p	3.99	3.80E-02	hsa-miR-4536-3p	33.67	1.61E-03	
hsa-miR-10396a-3p	4.28	2.51E-03	hsa-miR-203a-3p	35.11	1.02E-13	

FC mean from 5 samples is shown. sASC-EVs/ASC-EVs ratios with padj < 0.05 are shown. FC: Fold change; padj: adjusted *P*-value; ASC-EVs: adipose-derived stromal cell-derived extracellular vesicles; sASC-EVs: sorted adipose-derived stromal cell-derived extracellular vesicles; miRNA: microRNA; hsa: Homo sapiens.

Analysis of the 478 miRNAs exclusively identified in ASC-EVs revealed that, when their counts were examined across post-sorting samples [Figure 3E], all displayed at least three samples with zero counts, fulfilling the threshold for assignment as absent in sASC-EVs. Importantly, the majority of these miRNAs exhibited consistently low abundance across donors (Rlog < 6) [Figure 3F].

### Identification of pathways associated with identified EV miRNAs

**Figure 4:**
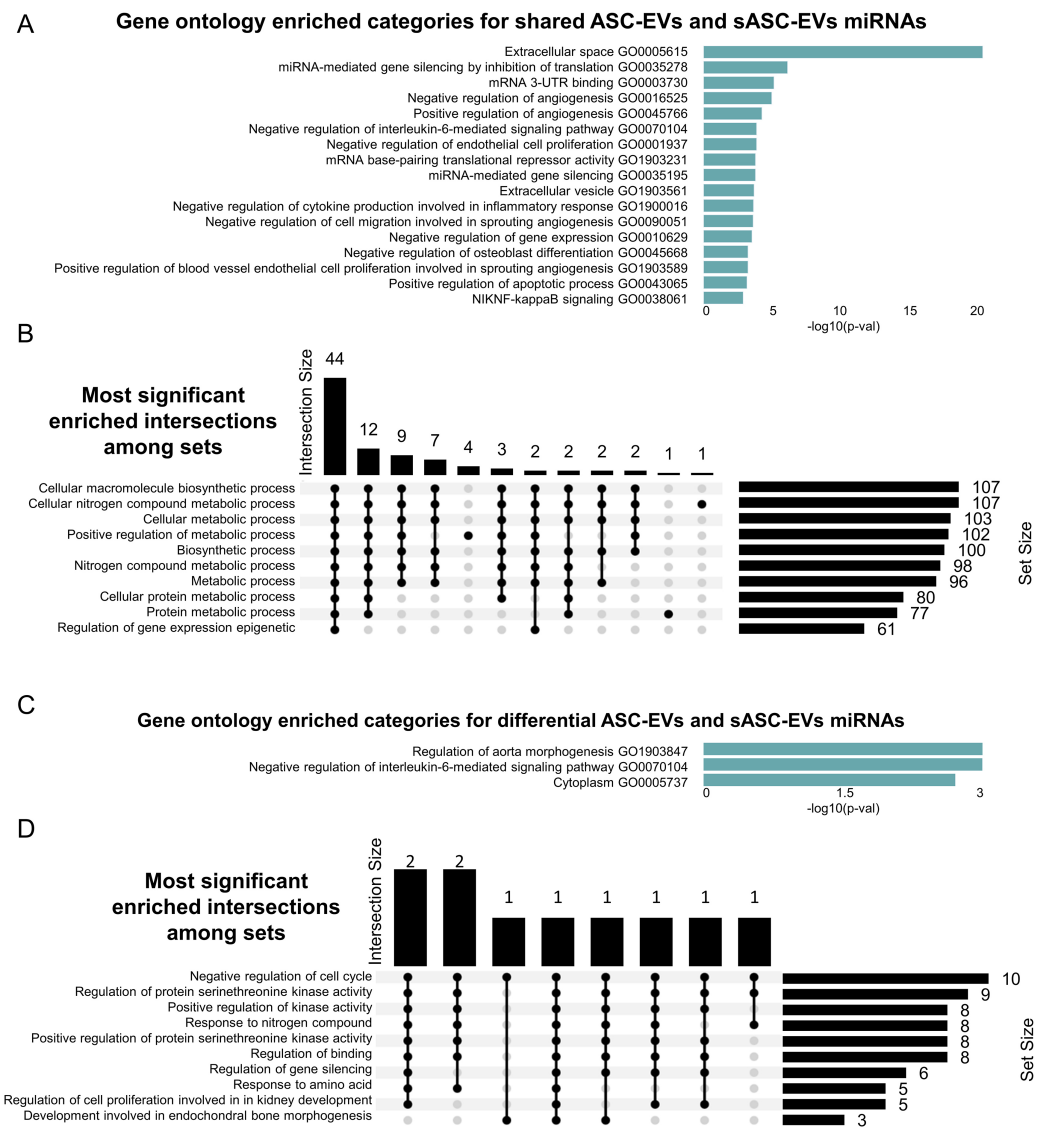
GO analysis of shared and differentially expressed miRNAs from ASC-EVs and sASC-EVs. (A) Bar chart of enriched GO categories for shared miRNAs, ranked by significance; (B) Upset plot showing the most significantly enriched intersections among sets. Horizontal bars on the left indicate the size of each individual set, while vertical bars represent the size of intersections between sets, as defined by the filled dots in the matrix. The height of each vertical bar corresponds to the number of elements in that intersection; (C) Bar chart of enriched GO categories for differentially expressed miRNAs, ranked by significance; (D) Upset plot of enriched intersections among sets for differentially expressed miRNAs, described as in (B). GO: Gene Ontology; miRNA: microRNA; ASC-EVs: adipose-derived stromal cell-derived extracellular vesicles; sASC-EVs: sorted adipose-derived stromal cell-derived extracellular vesicles.

*In silico* functional analysis of the 271 miRNAs shared between ASC-EVs and sASC-EVs was performed to identify GO categories and associated biological processes (BP) using the miEAA platform. GO enrichment analysis [Figure 4A and Supplementary Table 2] revealed several angiogenesis-related terms, including negative and positive regulation of angiogenesis (GO0016525/GO0045766), cell migration (GO0090051) and positive regulation of endothelial cell proliferation (GO1903589). The association with endothelial biology was further supported by the enrichment of negative regulation of cell proliferation (GO0001937). Additional terms indicated a strong influence on the extracellular space (GO0005615), encompassing inflammation-related processes such as the IL-6 signaling pathway (GO0070104) and negative regulation of cytokine production (GO1900016). Moreover, a broad impact on gene expression was predicted, highlighted by enrichment of miRNA-mediated gene silencing (GO0035278/GO0035195) and multiple categories of translational repression (GO0010629/GO0003730/GO1903231). These findings were further endorsed by the identification of several BP terms associated with metabolism and biosynthetic processes [Figure 4B].

Subsequent analyses focused on miRNAs differentially expressed between ASC-EVs and sASC-EVs. GO enrichment [Figure 4C and GO category (A) in Supplementary Table 3] yielded three significant terms, including confirmation of negative regulation of the IL-6-mediated signaling pathway (GO0070104). BP analysis [Figure 4D] demonstrated that these differentially expressed miRNAs may influence cell cycle regulation and kinase activity, thereby contributing to gene silencing. Reanalysis, including the sASC-EV-specific miRNAs (absent in ASC-EVs), reinforced these findings, again confirming IL-6 signaling-related GO terms [GO category (B) in Supplementary Table 3] and BP categories associated with cell cycle regulation and gene silencing (data not shown).

### Impact of EV miRNAs on OA pathology

To illustrate potential biological relevance of identified miRNAs in pathological settings, OA pathology was selected as a model disease to sift the 271 miRNAs shared between ASC-EVs and sASC-EVs, together with sASC-EV-specific players, due to a broad knowledge of miRNAs previously reported in the literature to exert protective, detrimental, or overlapping effects^[25,26,27]^. This analysis identified 31 protective, 20 detrimental and 12 overlapping miRNAs, plus one sASC-EV-specific miRNA [Table 3]. Within the protective group, seven miRNAs displayed high abundance (Rlog > 7), including the two most highly expressed ones, hsa-miR-24-3p and hsa-miR-125-5p. Using the same threshold, four of five detrimental miRNAs and three overlapping miRNAs also reached Rlog > 7. Overall, the ratio of protective to detrimental Rlog values was 1.9 for ASC-EVs and 1.8 for sASC-EVs, suggesting a favorable balance toward protective activity in both vesicle populations. When the analysis was restricted to the 38 differentially expressed miRNAs, four candidates of potential relevance to OA emerged. These included two protective miRNAs, hsa-miR-142-5p (3.8-fold change) and hsa-miR-222-5p (7.9-fold change), and two detrimental miRNAs, hsa-miR-138-5p (2.3-fold change) and hsa-miR-29b-3p (2.6-fold change). Notably, all four were expressed at relatively low levels, thereby corroborating the overall similarity of the protective-to-detrimental ratios between ASC-EVs and sASC-EVs.

**Table 3:** Role of shared EV miRNAs in OA

**Protective miRNA**	**ASC-EVs Rlog**	**sASC-EVs Rlog**	**Overlapping miRNA**	**ASC-EVs Rlog**	**sASC-EVs Rlog**	**Destructive miRNA**	**ASC-EVs Rlog**	**sASC-EVs Rlog**
hsa-miR-24-3p	12.2	11.8	hsa-miR-21-5p	9.8	10.1	hsa-miR-23a-3p	10.5	9.9
hsa-miR-125b-5p	12.2	12.1	hsa-miR-145-5p	9.8	9.2	hsa-miR-19b-3p	8.0	8.3
hsa-miR-199a-5p	9.3	9.6	hsa-miR-221-3p	7.8	7.7	hsa-miR-34a-5p	7.9	7.9
hsa-miR-29a-3p	8.9	9.3	hsa-miR-140-3p	5.9	6.4	hsa-miR-16-5p	6.9	7.0
hsa-miR-199a-3p	8.6	8.4	hsa-miR-98-5p	4.9	4.6	hsa-miR-455-3p	5.1	4.5
hsa-miR-92a-3p	8.4	8.0	hsa-miR-146a-5p	4.6	5.3	hsa-miR-21-3p	4.9	5.8
hsa-miR-222-3p	7.9	7.6	hsa-miR-27b-3p	4.5	4.1	hsa-miR-186-5p	4.9	5.0
hsa-miR-27a-3p	7.2	7.6	hsa-miR-26a-5p	4.3	4.1	hsa-miR-324-5p	4.6	4.0
hsa-miR-210-3p	6.8	6.7	hsa-miR-140-5p	4.2	3.9	**hsa-miR-29b-3p**	**4.3**	**6.0**
hsa-miR-335-5p	6.7	6.0	hsa-miR-145-3p	3.7	3.5	hsa-miR-101-3p	4.2	5.0
hsa-miR-130a-3p	6.5	7.4	hsa-miR-98-3p	2.3	1.8	hsa-miR-181a-5p	4.1	4.2
hsa-miR-93-5p	6.1	5.5	hsa-miR-15a-5p	1.4	2.3	**hsa-miR-138-5p**	**3.8**	**4.9**
hsa-miR-19a-3p	6.1	5.8	hsa-miR-9-3p*	/	1.1	hsa-miR-29c-3p	3.4	2.9
hsa-miR-17-5p	5.5	5.6	TOTAL	63	63	hsa-miR-34a-3p	3.3	3.3
hsa-miR-152-3p	5.3	5.7	* sASC-EVs			hsa-miR-30b-5p	3.2	3.9
hsa-miR-103a-3p	5.2	4.8				hsa-miR-181b-5p	2.6	2.6
hsa-miR-193b-3p	5.2	4.1				hsa-miR-221-5p	2.6	3.2
hsa-miR-193b-5p	5.1	4.6				hsa-miR-23a-5p	2.6	2.7
hsa-miR-148a-3p	5.0	5.5				hsa-miR-381-3p	1.4	1.5
hsa-miR-337-3p	4.9	4.1				hsa-miR-223-3p	0.3	2.5
hsa-miR-377-3p	4.1	4.2				Total	89	95
hsa-miR-30a-3p	3.6	2.5						
hsa-miR-329-3p	3.0	3.1						
hsa-miR-149-5p	2.9	3.6						
hsa-miR-197-3p	2.8	3.6						
hsa-miR-26b-5p	2.6	2.8						
hsa-miR-195-5p	2.4	2.6						
**hsa-miR-142-5p**	**0.9**	**2.6**						
hsa-miR-188-5p	0.8	1.6						
hsa-miR-142-3p	0.8	1.8						
**hsa-miR-222-5p**	**0.4**	**2.1**						
TOTAL	167	171						
PROT/DES	1.9	1.8						

In bold, sASC-EVs upregulated miRNAs. Rlog mean of five samples is shown. EVs: Extracellular vesicles; ASC-EVs: adipose-derived stromal cell-derived extracellular vesicles; sASC-EVs: sorted adipose-derived stromal cell-derived extracellular vesicles; miRNA: microRNA; OA: osteoarthritis; Rlog: regularized log transformation; hsa: Homo sapiens; PROT/DES: protective to destructive miRNA ratio.

## DISCUSSION

In this proof-of-concept study, we performed a comparative analysis of miRNA cargo of EVs derived from adipose-derived mesenchymal stromal cells (ASCs), before and after fluorescence-based sorting (ASC-EVs and sASC-EVs, respectively). Although the overall size distribution, morphology and immunophenotypic profiles of the EVs remained consistent, a markedly lower number of miRNAs were detected in the sASC-EV samples. This observation is notable considering the robust correlation of abundance for shared miRNAs and the preservation of key biological signatures post-sorting, yet it raises important questions regarding the origin and significance of the miRNAs lost during the sorting process.

A possible explanation for the reduced miRNA heterogeneity observed in sASC-EVs relates to the efficiency of quantitative recovery of initial EVs following sorting. Despite the retention of consistent immunophenotypic and size features across replicates, flow cytometry-based sorting may inherently result in a substantial loss of particles, particularly when nanometer-sized^[28]^, and in our workflow, only 10% of the initially sorted EVs were effectively recovered. Given the relatively low throughput of this and other cytometric platforms for nanoparticles, the overall amount of encapsulated RNA recovered, and consequently the sequencing depth for rare miRNAs, was likely diminished, as also supported by the markedly lower number of assigned reads (17%) in the sASC-EV samples compared to pre-sorted samples, considering that both sample types were derived from identical initial volumes of cell-culture supernatant and thus almost 90% fewer EVs were processed for RNA extraction and NGS in sASC-EVs. It is therefore possible that many miRNAs appearing as lost after sorting do not reflect their true biological absence but rather a technical limitation arising from under-sampling, since the low RNA yield inevitably biases the analysis towards the most abundant species; accordingly, the apparent reduction in miRNA complexity following sorting should be interpreted with caution, as it may reflect technical rather than purely biological differences.

In addition to efficiency issues affecting overall EV recovery, other technical limitations of the flow cytometer used for nanoparticle sorting may have contributed to the selective exclusion of EVs below ~100 nm in diameter. In fact, the diffraction limit fundamentally restricts the resolution achievable when imaging smaller cells and particles^[29]^. While flow cytometry excels at rapid analysis of large cell populations, achieving true diffraction-limited resolution at high throughput remains a challenge. This limit, determined by the wavelength of light and the numerical aperture of the objective lens, dictates the smallest feature size that can be clearly resolved. Despite the protocol used for EV sorting allowing detection of particles smaller than 200 nm^[13]^, vesicles approaching or below this size can still be poorly resolved or even undetectable^[30]^. Most conventional flow cytometers have a lower detection limit between 300 and 500 nm, although optimized triggering strategies, as applied in our study, can extend detection down to approximately 100 nm^[31-33]^. As a result, the smaller EVs may remain unsorted. These smaller EVs may differ in cargo content^[34,35]^, including miRNA profiles, and their underrepresentation in sASC-EVs could skew the perceived diversity. Nevertheless, NTA of our dataset revealed no differences in EV size or distribution profiles between pre- and post-sorted samples, suggesting that this limitation likely had a minimal impact. Moreover, the high correlation in abundance among shared miRNAs, together with similar expression patterns for the most enriched candidates in both EV types, supports the hypothesis that core, high-abundance miRNAs are robustly captured and detected across the two conditions, whereas low-abundance, size-associated, or co-isolated contaminants are disproportionately lost post-sorting.

In this context, the potential exclusion of contaminating non-vesicular nanoparticles present in pre-sorted samples may also contribute to the broader miRNA repertoire observed in ASC-EVs. These entities - including protein complexes, lipoproteins, and ribonucleoprotein aggregates - are known to carry extracellular miRNAs independent of membrane-enclosed vesicles^[36,37]^. Flow cytometric sorting, selectively enriching bona fide EVs, should greatly reduce such contaminants with respect to other approaches, such as ultracentrifugation without previous purification steps, where contaminants are co-isolated with EVs^[38]^. Therefore, this “cleaning” effect, while improving specificity, could also eliminate a portion of associated nucleic acids not truly vesicular in origin, explaining the loss of low-abundance miRNAs in the sorted preparations further processed by ultracentrifugation before NGS.

Interestingly, despite the lower number of detected miRNAs and their high overall similarity, sASC-EVs displayed downregulation of 6 and upregulation of 32 miRNAs, alongside the presence of 14 specific players (although with low abundance). The disproportion in favor of miRNA increase further supports one of our previous hypotheses: that sorting either selectively enriches vesicle subpopulations with specific miRNA signatures or reduces dilution by non-vesicular sources, thereby decreasing the relative abundance of certain elements lost during sorting. Despite these differences, functional enrichment analysis confirmed the overall retention of relevant biological signals, particularly those related to IL-6 signaling, angiogenesis and gene silencing, all of which are pertinent to tissue repair and inflammation regulation. Considerably, these features of ASC-EVs are in agreement with several reports describing their therapeutic role in wound healing and tissue regeneration^[39]^, extracellular matrix remodeling^[40]^ and angiogenesis^[41]^, with a broad application in regenerative medicine approaches^[42]^ such as OA treatment^[43]^.

In this study, the miRNA cargo shared between ASC-EVs and sASC-EVs highlighted relevance to OA pathophysiology, used here as a model disease to highlight future translational investigations of EV sorting-sequencing technology. Functionally, enrichment of negative regulators of cytokine production and IL-6-mediated signaling is notable, given the central role of IL-6 in synovial inflammation^[44]^, cartilage catabolism^[45]^ and pain^[46]^. These data support an anti-inflammatory effect of ASC-EV miRNAs on OA chondrocytes and synoviocytes^[47]^. Similarly, regulation of gene silencing and translational repression aligns with reported ASC-EV effects on OA, including modulation of Wingless/Integrated (WNT) signaling, a key pathological pathway and therapeutic target^[48]^. For instance, hsa-miR-376c-3p, present among shared miRNAs, can repress WNT-β-catenin signaling by targeting *WNT3* or *WNT9*^[49]^, with *WNT3* also potentially targeted by hsa-miR-181a-5p, hsa-miR-16-5p, hsa-miR-15a-5p, hsa-miR-128-3p and hsa-miR-103a-3p, as well as other WNT family members (*WNT1/2/7/10*). Comparison with literature-reported OA-associated miRNAs further supports the relevance of the shared EV cargo. Protective miRNAs predominated, with protective-to-detrimental Rlog ratios of 1.9 and 1.8 for ASC-EVs and sASC-EVs, respectively. Among these, hsa-miR-24-3p and hsa-miR-125b-5p were the most abundant, consistent with prior real-time reverse transcription PCR (qRT-PCR) results from ultracentrifuged ASC-EVs^[50]^. hsa-miR-24-3p is downregulated in OA^[51]^, reduces metalloproteinase secretion, modulates cartilage catabolism^[52]^ and promotes M2 macrophage polarization^[53]^, whereas hsa-miR-125b-5p inhibits proinflammatory cytokines and matrix destruction in human OA chondrocytes^[54]^ and attenuates inflammation and apoptosis in OA models^[55]^. The maintained abundance of these miRNAs and the favorable protective-to-detrimental ratio after sorting underscores the preserved therapeutic potential of ASC-EVs.

Several limitations of this study arise from its nature as a proof-of-concept investigation. First, only five ASC-EV donors were included, primarily due to the technically demanding, time-consuming and costly nature of the fluorescence-based sorting protocol. Moreover, all donors were female and of approximately the same age, which introduces both gender and age bias. While this relatively homogeneous group was intentionally chosen to minimize inter-donor variability and establish technical feasibility, the findings cannot be generalized to broader, more diverse populations. This limitation reflects the focus on providing a proof-of-concept regarding the impact of sorting on the EV miRNA repertoire, rather than offering a definitive roadmap for the MSC-EV miRNA fingerprint. Future studies with larger and demographically varied cohorts are essential to validate these results and fully assess the biological relevance of ASC-EVs across different donor backgrounds. Second, the low-throughput nature of the sorting technique limited the number of EVs subjected to NGS [on the order of 10^6^ particles], resulting in reduced RNA yield and overall miRNA detection. It is not yet clear whether the missing miRNAs are due to the removal of “environmental” miRNAs potentially present in unsorted RNA, or if this is the result of technical artefacts introduced during EV sorting. Nevertheless, since EVs share the same size distribution and surface markers, it seems unlikely that biological selection occurs during sorting. Instead, the low recovery and reduced RNA yield might explain the lower complexity and the preferential detection of abundant miRNAs. To address this issue, we plan to improve our protocol to enhance sorting efficiency and thereby validate and clarify these findings. In fact, due to the limited yield of EVs recovered after sorting, the material was sufficient only for sequencing analysis but did not allow further validation using orthogonal methods. Nevertheless, the sequencing data were internally consistent, with strong correlation of shared miRNA abundance between ASC-EVs and sASC-EVs and reproducibility across donors. In addition, both the abundance of key miRNAs, including hsa-miR-24-3p and hsa-miR-125b-5p, is supported by previous results of our group obtained with qRT-PCR technology on independent ASC-EVs samples^[56]^, reinforcing the functional relevance of the observed patterns. Future studies with higher EV yields will enable independent validation of differentially expressed miRNAs while preserving the proof-of-concept focus on sorting technology of this study. Third, due to the relatively large post-sorting volumes (5-10 mL), ultracentrifugation was required to concentrate EVs for RNA extraction prior to sequencing. This additional step may introduce bias into the resulting miRNA profiles. As such, the current sorting approach may be more suitable as a purification step prior to other isolation or enrichment techniques, rather than a standalone method. Further technical improvements are needed to enable direct RNA analysis from more concentrated and minimally manipulated sorted samples. Lastly, OA-related miRNAs were classified based on two up-to-date reviews, assigning intermediate or overlapping roles in cases of conflicting evidence. We acknowledge that the literature on OA is broader, quickly evolving and highly context-dependent, with new players discovered on a regular basis and many miRNAs reported as both protective and detrimental under different conditions. Importantly, OA was used here as a model disease, and the primary aim of this study was to evaluate the impact of sorting on miRNA detection and abundance, serving as a proof-of-concept for the technology, rather than providing a definitive functional annotation, which will require broader literature integration and functional validation in future studies. Despite these limitations, the present work provides a methodological foundation for advancing fluorescence-based EV sorting and lays the groundwork for subsequent and pathology-focused translational investigations.

Taken together, our findings highlight the trade-off between EV purity and molecular diversity inherent in sorting procedures. While flow cytometry allows for precise selection of EVs, it may exclude subsets of vesicles or co-isolated complexes harboring biologically meaningful miRNAs. These results emphasize the importance of standardizing EV purification protocols according to the intended downstream application, particularly for biomarker discovery or therapeutic use, where either molecular complexity or sample purity may take precedence. Future improvements in high-resolution sorting technologies and multimodal EV profiling will be critical for disentangling the contributions of vesicle subtypes and co-isolated structures to the observed miRNA repertoire, ultimately enhancing our understanding of EV biology and their role in disease modulation. At present, sorting technology may serve as a useful intermediate step to remove unwanted contaminants, with the anticipation that future high-throughput protocols will enable recovery of large numbers of pure EV populations or subpopulations with enhanced therapeutic potential.

## DECLARATIONS

### Availability of data and materials

Raw data are available at: https://osf.io/hfjcu/?view_only=f6c3ffd8616d4646b28ba9b446e0e93c.

### AI and AI-assisted tools statement

Artificial intelligence assistance was used in the preparation of this manuscript for language editing.

### Copyright

© The Author(s) 2026.
